# Potato Chips Byproducts as Feedstocks for Developing Active Starch-Based Films with Potential for Cheese Packaging

**DOI:** 10.3390/foods12061167

**Published:** 2023-03-09

**Authors:** Ana M. Peixoto, Sílvia Petronilho, M. Rosário Domingues, Fernando M. Nunes, Joana Lopes, Marit Kvalvåg Pettersen, Magnhild S. Grøvlen, Elin M. Wetterhus, Idalina Gonçalves, Manuel A. Coimbra

**Affiliations:** 1 LAQV-REQUIMTE—Associated Laboratory for Green Chemistry of the Network of Chemistry and Technology, Department of Chemistry, Campus Universitário de Santiago, University of Aveiro, 3810-193 Aveiro, Portugalmac@ua.pt (M.A.C.); 2 Chemistry Research Centre-Vila Real, Department of Chemistry, University of Trás os-Montes and Alto Douro, Quinta de Prados, 5001-801 Vila Real, Portugal; 3 Mass Spectrometry Centre, Department of Chemistry, Campus Universitário de Santiago, University of Aveiro, 3810-193 Aveiro, Portugal; 4 CESAM—Centre for Environmental and Marine Studies, Department of Chemistry, Campus Universitário de Santiago, University of Aveiro, 3810-193 Aveiro, Portugal; 5 CICECO—Aveiro Institute of Materials, Department of Materials and Ceramic Engineering, Campus Universitário de Santiago, University of Aveiro, 3810-193 Aveiro, Portugal; 6 Nofima—Norwegian Institute of Food, Fisheries and Aquaculture Research, NO-1431 Ås, Norway

**Keywords:** potato frying residue, melanoidins, starch films, active packaging, volatile oxidation products, circular economy

## Abstract

The potato chip industry generates brownish frying residues, which are usually landfilled. While spent frying oil has value as biodiesel, the defatted brownish water-soluble extract (BrE) does not yet have an application. In this work, it was hypothesized that BrE can be a source of compounds for active packaging. BrE is composed of carbohydrates (66.9%), protein (5.7%), and a small amount of phenolics and esterified fatty acids. When incorporated into starch-based formulations and casted, BrE at 5%, 10%, and 15% *w*/*w* (dry starch weight) conferred a yellowish coloration while maintaining the transparency of neat films. The BrE increased the films’ traction resistance, elasticity, and antioxidant activity while decreasing their hydrophilicity. Furthermore, starch/15% BrE-based films showed diminished water vapor and good UV-light barrier properties. Their contact with sliced cheese did not change the products’ hardness during storage (14 days). Weight loss of the cheese was observed after 7 days of storage, stabilizing at 6.52%, contrary to the cheese packed in polyamide (PA)/polyethylene (PE), already used in food packaging. The cheese packed in the starch/15% BrE-based films showed a significant yellowish darkening and lower content of volatile oxidation products compared to the PA/PE. Therefore, BrE revealed to have compounds with the potential to tune the performance of starch-based films for food packaging.

## 1. Introduction

Potato (*Solanum tuberosum* L.) is one of the most widely cultivated and consumed carbohydrate-rich food crop worldwide, with a global production of ca. 359 million tons in 2020 [1]. Although they can be eaten in different ways, potato chips are one of the most widely appreciated and consumed potato product due to the fact of their characteristic salty flavor and crispy texture [2,3]. Their industrial frying process involves multiple steps, including washing, peeling, slicing, and frying in fat or vegetable oil at high temperatures [2]. Several tons of potato byproducts are generated, including washing slurries, peels, spent frying oil, and a brownish frying residue present in the frying oil. This residue is composed of burned potatoes accumulated at the bottom of noncontinuous friers, usually used in small and medium industries and household and restaurant kitchens. The valorization of these byproducts as raw materials to create added-value products is under research, promoting a more natural, low-cost, and sustainable production while encouraging a circular economy [4].

Potato washing slurries have been used to recover starch to develop bioplastics with physicochemical and barrier properties competitive with the ones provided by commercial potato starch [5]. To improve the elastic and hydrophobic properties of starch-based films, spent frying oil [6] and waxes recovered from potato peels [5] have been used. In addition, potato peel phenolic extracts [7] have demonstrated the potential to improve the antioxidant activity and UV-light barrier properties of starch-based films. While the frying oil is channeled for biodiesel production [8], at present, the defatted brownish water-soluble extract (BrE), a putative source of high molecular weight nitrogenous, brown-colored compounds (i.e., melanoidins), is a disposable industrial byproduct that originates environmental issues.

Due to the frying conditions of potatoes (high-temperature and anhydrous environment), melanoidins can be formed during nonenzymatic browning reactions (i.e., Maillard reaction) involving compounds with amino and carbonyl groups [2,9]. Moreover, the fatty acids of triacylglycerides present in the frying oil may be incorporated into the potato chips’ melanoidins’ structure, similar to what has been observed for other organic acids in coffee melanoidins [10]. Despite these assumptions, the chemical composition of BrE remains unknown. Since melanoidins from thermally processed food have shown bioactive potential [11,12], they should be exploited for different applications, such as in the development of active bio-based materials with potential for food packaging, similar to what has been conducted with potato peels and starch [7].

In this work, it was hypothesized that BrE, when gelatinized with starch recovered from potato washing slurries and casted, can give rise to bioplastics that can be used as active packaging to preserve sliced cheese characteristics, a foodstuff susceptible to lipid oxidation. For this, a hot-water extraction was applied to the defatted brownish frying residue. The influence of BrE on the optical, mechanical, wettability, water solubility, water vapor transmission rate, and active (UV-protective and antioxidant) properties of potato starch-based films was evaluated. The suitability of using starch/BrE-based films as active packaging was assessed in sliced cheese.

## 2. Materials and Methods

### 2.1. Materials

The potato washing slurries and potato chip frying residue were supplied by the A Saloinha Lda. company (Mafra, Portugal). Starch (25% amylose, 59–71 °C gelatinization temperature, and 12.5 J/g enthalpy) was recovered from the lyophilized slurries [5]. The brownish water-soluble extract (BrE, Figure S1) was obtained after defatting (spent frying oil recovered by Soxhlet extraction with a chloroform/methanol (2:1, *v*/*v*) mixture), hot-water extraction (80 °C, 1 h, constant stirring), centrifugation (24,000× *g*, 4 °C, 20 min), and freeze-drying. The BrE was stored in a desiccator containing phosphorus pentoxide until further characterization and usage in the films’ production. Glycerol was purchased from Scharlab S.L. (Barcelona, Spain), and the sodium azide, chloroform and methanol were acquired from Sigma-Aldrich (Lisbon, Portugal). All reagents were of analytical grade and used without purification.

### 2.2. Fractionation of Potato Chip Brownish Water-Soluble Extract (BrE)

**Figure 1:**
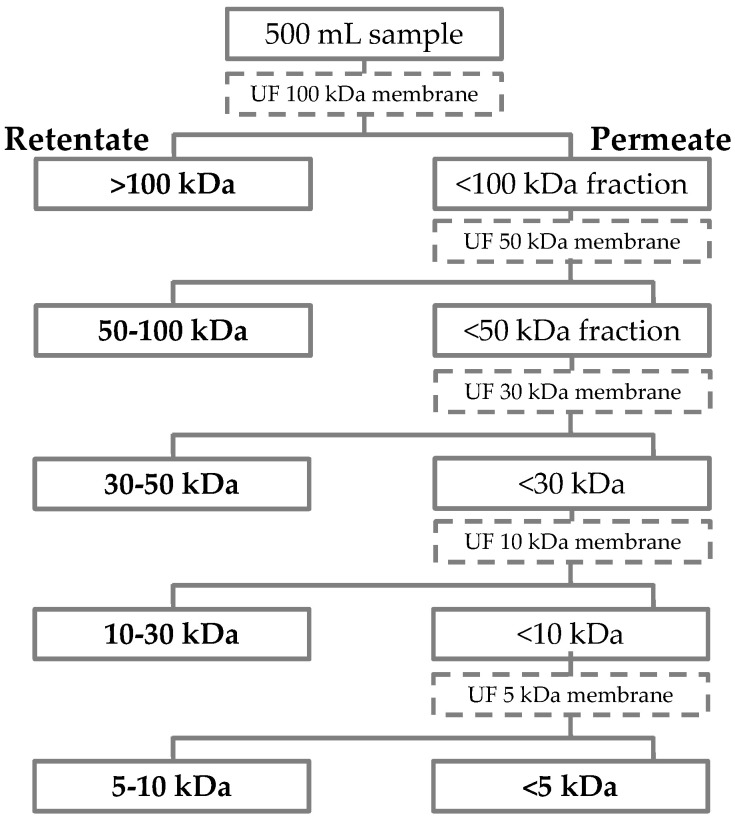
Scheme of the ultrafiltration process used for the BrE fractionation using 100, 50, 30, 10, and 5 kDa cut-off membranes.

The BrE was submitted to a fractionation procedure by ultrafiltration (UF) using the Millipore Labscale™ TFF System equipped with a 500 mL reservoir [13]. Five cut-off membranes were used (100, 50, 30, 10, and 5 kDa from Merck Millipore Pellicon XL 50 cassettes with a flat plate format) at room temperature (20 °C ± 2 °C) and working between 10 to 30 psi transmembrane pressures. The separation efficiency was controlled by measuring the permeate conductivity (until it presented a value below 10 μS/cm) and determining the UV–Vis absorption (420 nm and 405 nm to monitor the presence of brown-colored compounds and 325 nm and 280 nm for phenolics and proteins). The ultrafiltration process was repeated for 3 cycles for each membrane (Figure S2). At the end of the procedure, 6 fractions corresponding to the >100 kDa, 50–100 kDa, 30–50 kDa, 10–30 kDa, and 5–10 kDa retentates, and the <5 kDa permeate were recovered. Figure 1 illustrates the flowchart to obtain each fraction according to the relative molecular weight. All UF fractions were frozen, freeze-dried, and stored under an anhydrous atmosphere until further characterization.

### 2.3. Characterization of Potato Chip Brownish Water-Soluble Extract (BrE)

The sugar composition and content of the BrE and correspondent UF fractions were determined, in triplicate, by gas chromatography with flame-ionization detection (GC-FID) as alditol acetates. The 2-deoxyglucose was used as the internal standard [14].

The protein content of the BrE and UF fractions was estimated, in triplicate, by determining the total nitrogen in a Truspec 630-200-200 elemental analyzer (St. Joseph, Berrien, MI, USA) with a thermal conductivity detector (TDC). The nitrogen content was converted into protein content (%, *w*/*w* extract) using the 6.25 factor [15].

The total phenolic content of the BrE and UF fractions was determined, in triplicate, using the Folin–Ciocalteu method, where the absorbance of the samples was measured at 750 nm. A calibration curve of gallic acid (50–250 μg/mL) was used, and the results were expressed as μg gallic acid equivalents/mg of extract (μg GAE/mg) [12].

The esterified fatty acids (EFAs) content was determined by GC-FID, in triplicate, as fatty acid methyl esters (FAMEs) after the alkaline-catalyzed transesterification of the esterified fatty acids of the sample. For this, heptadecanoate methyl ester (0.03 mg/mL prepared in n-hexane) and KOH methanolic solution (2M) were used [6]. A DB-FFAP column (30 m × 0.32 mm and 0.25 μm of film thickness, J&W Scientific Inc., Folsom, CA, USA) was used. The compounds were identified by comparing their retention times with those of a commercial FAME mixture (C_8_–C_24_).

The presence of melanoidins was assessed by determining the specific extinction coefficient (K_mix_) and the melanoidin browning index (MBI). The K_mix_ was determined at 405 nm (K_mix 405nm_) to determine the relative content of the brown-colored compounds in each UF fraction [14,16]. Briefly, for each sample, different solutions were prepared in distilled water (0.1 to 1 mg/mL), and the absorbance was measured, in triplicate, at room temperature (20 °C ± 2 °C). The MBI was calculated by dividing the K_mix 405nm_ value by the relative content of the unknown material estimated by the difference in the known one (sugars and protein) [12,17].

The antioxidant activity of each sample was determined, in triplicate, using the ABTS (2,2′-azino-bis(3-ethylbenzothiazoline-6-sulphonic acid)) assay [18] and expressed as IC_50_ (minimal concentration of extract required for 50% inhibition of ABTS^●+^). Briefly, 50 μL of each sample was added to 250 μL of 7 mM ABTS solution. The absorbance was read after 20 min at 734 nm using a microplate spectrophotometer (BioTech™, Eon™, Richmond Scientific, Lancashire, Great Britain). Water was used as a blank, ABTS solution as the control, and ascorbic acid as the standard [12].

### 2.4. Production of Starch/BrE-Based Films by Solvent Casting

The starch/BrE-based films were produced by solvent casting following a previously described method [6], with the addition of the BrE at different proportions (5%, 10%, and 15% *w*/*w*, starch dry weight). Briefly, a water dispersion of freeze-dried potato starch (40 g/L) was prepared, and 12 g/L of glycerol was added as well as BrE in different proportions (2 g/L, 4 g/L, and 6 g/L. Then, each dispersion was gelatinized at 95 °C ± 0.1 for 30 min with constant stirring (ca. 200 rpm). The potato starch-based films without BrE (neat films) were used as a control.

### 2.5. Characterization of the Starch/BrE-Based Films

#### 2.5.1. Chromatic Properties

The chromatic properties of the films were assessed by tristimulus colorimetry (CIELab). The CIELab coordinates L* (luminosity), b* (yellow/blue), and a* (red/green) were determined using a CR-400 Chroma Meter. The total color difference (ΔE) was also assessed [6].

#### 2.5.2. Thickness and Mechanical Properties

The mechanical properties of the films were determined using a texture analyzer (model TA. Hdi, Stable Micro Systems, Surrey, UK). Each film formulation was cut (12 strips for each film sample, each one a size of 1 cm × 9 cm), and the thickness was measured in 3 different strip points with a digital micrometer (ca. 0.001 mm accuracy; Mitutoyo Corporation, Kanagawa, Japan). During the uniaxial tensile tests till film failure, the tensile strength (MPa), elongation at break (%), and Young’s modulus (Mpa) values were determined from the stress–strain curves provided by Exponent software [6].

#### 2.5.3. Wettability

The wettability of each film was determined at room temperature (20 °C ± 2 °C) through static water contact angles (WCAs) using a tensiometer (Attention Theta by Biolin Scientific, Madrid, Spain) fitted with an automatic image capture system (One Attension). Each formulation was cut in strips (1 cm × 6 cm) and tested in triplicate for both film surfaces: one exposed to air (film upper surface) and one in contact with a plexiglass plate (film’s lower surface). Briefly, 3 μL of ultrapure water were dispensed on the film strips’ surfaces, and the corresponding WCAs (at least 6 droplet images along each strip) were measured using One Attention software following the Young–Laplace method [6].

#### 2.5.4. Water Solubility

To determine the films’ solubility in water, squares (4 cm^2^) of each film formulation were cut, weighed, and immersed into distilled water containing sodium azide (0.02% *v*/*v*) at room temperature (20 °C ± 2 °C) at 80 rpm for 14 days. The non-solubilized films were dried (105 °C, overnight), cooled down to room temperature, and weighed. The solubility of each sample was evaluated as a weight loss percentage, in triplicate [6]. The possible migration of the BrE components to the water during the solubility assays was evaluated by UV–Vis (200 to 700 nm), in triplicate, at the end of the assay.

#### 2.5.5. Water Vapor Transmission Rate

The water vapor transmission rate (WVTR) of the films (28 mm diameter per sample) was assessed, in triplicate, using test dishes and a humidity chamber (53% relative humidity) maintained at 23 ± 2 °C and with ca. 160 m/min of air velocity [6].

#### 2.5.6. UV-Protective and Antioxidant Activity

The UV-protective capacity of the films was monitored through a UV–visible spectrophotometer (Shimadzu UV 1280) from 200 to 500 nm. For each spectrum, a representative average of 3 scans was obtained using a data pitch of 0.5 nm, a bandwidth of 2.0 nm, and a scanning speed of 100 nm/min [19].

The antioxidant activity of the films was determined by an adaptation of the ABTS method, as already described [19]. Briefly, each film square (4 cm^2^) was placed in 1.5 mL ABTS^•+^ solution (7 mM ABTS in 2.45 mM potassium persulfate) and left to react under dark conditions (20 °C ± 2 °C, 80 rpm). The absorbance at 734 nm was measured for 4 h. An ABTS^•+^ solution without film was used as a blank. The antioxidant activity was determined, in triplicate, as the percentage of ABTS^•+^ inhibition.

### 2.6. Sliced Cheese Package with Starch/15% BrE-Based Films

A commercial sliced cheese (Norvegia^®^ Original, semi-hard matured cheese, 27% fat, valid until 22 November 2022, storage 0–4 °C, L6 17.27, TINE SA, Norway) was packed using the starch/15% BrE-based films. A total of 4 cheese slices were sealed with the films (31 cm × 21 cm) using a sealing machine (Packer^®^, Packer Poly Sealer, King’s Lynn, Norfolk, UK). The packed samples were stored at 4 °C at 80% relative humidity for 14 days. A commercially available plastic of 90 µm polyamide/polyethylene (70 µm PA/20 µm PE, Allfo Vakuumverpackungen, Waltenhofen, Germany) was used as a control. After 7 and 14 days, the cheese samples were evaluated in terms of weight loss, the color CIELab coordinates L*, a*, and b* were determined using a CR-400 Chroma Meter (Konica Minolta, Inc, NJ, USA), as well as the texture (texture analyzer model TA. Hdi, Stable Micro Systems equipped with a 6 mm cylinder stainless probe for puncture tests [20]). The volatile profile of the cheese samples was analyzed by headspace-gas chromatography-mass spectrometry (HS-GC-MS) [21], where ethyl heptanoate (~98% purity, Fluka, Steinheim, Germany) was used as the internal standard, and the results are expressed as μg ethyl heptanoate equivalents (eq.)/g of the sample. The results are reported as the average of three independent replicates per each sampling moment.

### 2.7. Statistical Analysis

The results were statistically evaluated using the Student’s *t*-test with a significance level of 95% and *p* < 0.05, using the “*t*-test” tool in Excel 2016. Moreover, for multiple comparison analysis, one-way ANOVA with 95% probability level was used using GraphPad Prism version 8 for Windows (trial version GraphPad software, San Diego, CA, USA).

## 3. Results and Discussion

The defatted potato chip sample used in this work corresponded to 58% *w*/*w* of the potato chips frying residue supplied by the industry (Figure S1) [6]. From this, the brownish hot water-soluble extract (BrE) corresponded to 57.2% *w*/*w* concerning the defatted residue (Table 1). The BrE was fractionated by ultrafiltration (UF) and characterized in terms of carbohydrates, protein, lipids, and phenolic content. Moreover, the presence of melanoidins in the UF fractions was estimated, as well as their antioxidant activity.

### 3.1. Characterization of the Brownish Water-Soluble Extract (BrE)

The BrE was mainly composed of carbohydrates (66.9% *w*/*w*), with glucose, the structural unit of potato starch, as the major constituent (93.9 mol%). Galactose and arabinose, probably from arabinogalactan proteins [22], were also determined in this brownish extract, but in smaller amounts (5.20 mol% and 0.95 mol%, respectively). The BrE also presented protein (5.70% *w*/*w*) and a small amount of phenolics and lipids (ca. 0.40% and ca. 0.10% *w*/*w*, respectively).

The BrE was fractionated by ultrafiltration using five cut-off membranes, followed by freeze-drying. A total of six fractions, corresponding to the >100 kDa, 50–100 kDa, 30–50 kDa, 10–30 kDa, and 5–10 kDa retentates and to the <5 kDa permeate, were obtained. Since the yield of the fractions 30–50 kDa, 10–30 kDa, and 5–10 kDa only represented a total of ca. 3% *w*/*w* of BrE (Figure S3), only the fractions with a higher yield (>100 kDa, 50–100 kDa, and <5 kDa) were chemically characterized (Table 1).

**Figure 2:**
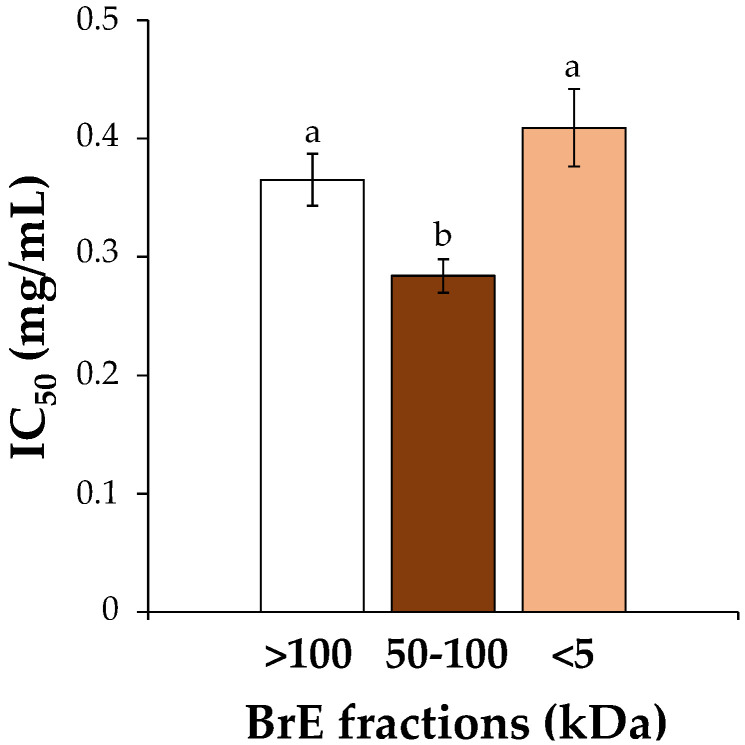
Antioxidant activity, expressed as IC_50_ values, determined for each BrE fraction. Different colors represent the chromatic tone of each fraction. The values that are significantly different (*p* < 0.05) are represented by different lowercase letters.

The major fraction (>100 kDa, 49.3% of BrE weight) was very rich in carbohydrates (89.4%), mainly glucose from starch, with a small portion of galactose and arabinose (Table 1). Protein accounted for 7.6%. The second largest fraction (<5 kDa, 39.9% of BrE weight) consisted of 37.1% sugars, with 10.0% accounting for free sugars and 16.4% protein. The presence of minerals can explain the remaining material [23]. Although not relevant in terms of yield (3.30% of BrE weight), the 50–100 kDa fraction was also different from the others due to the fact of its intense brown color, measured at 405 nm (K_mix,405 nm_ = 0.69 L/g/cm). Carbohydrates accounted for 29.4%, the protein was 17.4%, and the phenolics were 7.20%. Esterified fatty acids accounted for only 0.16%, mainly constituted by oleic (53.7%) and palmitic (36.3%) acid residues derived from spent frying oils due to the fact of their similar proportions [6]. These values allowed to estimate the amount of unknown material of 53.20%, corresponding to a melanoidin browning index (MBI) of 1.48. This value is not as high as the MBI value of 2.44 determined for the 30–100 kDa fraction of instant coffee [17]. In instant coffee, the >100 kDa fraction presented the highest MBI (3.31), not verified for BrE fraction > 100 kDa. This can be explained by the high content of starch in the potato present in the >100 kDa fraction and the high content of protein in the 50–100 kDa fraction, which should have promoted the Maillard reactions with carbohydrates [10,12]. In addition, a significantly higher antioxidant activity was determined in 50–100 kDa fraction (IC_50_ = 0.28 mg/mL, Figure 2), which can have a contribution of melanoidins [11], although the most abundant fractions also present a relevant antioxidant activity. This value was consistent with the IC_50_ value determined for chicory melanoidins (IC_50_ = 0.28 mg/mL) and higher than the one for instant coffee melanoidins (IC_50_ = 0.08 mg/mL) [12]. Due to the low yield of the 50–100 kDa fraction, the unfractionated BrE was used for the preparation of potato starch-based films.

### 3.2. Characterization of the Potato Starch/BrE-Based Films

#### 3.2.1. Chromatic Properties

**Figure 3:**
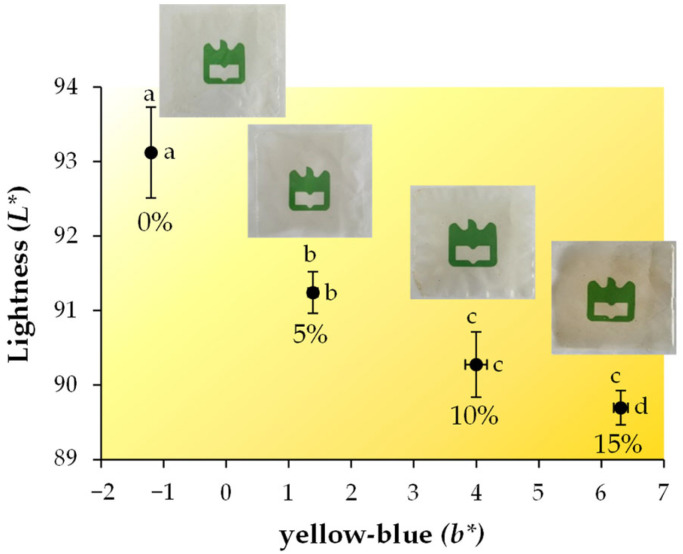
Images and L* and b* CIELab coordinates of potato starch-based films containing different amounts of BrE (0%, 5%, 10%, and 15% *w*/*w* of dry starch weight). The values that are significantly different (*p* < 0.05) are represented by different lowercase letters.

The neat films exhibited L*, a*, and b* values of 93.1, 1.73, and −1.20, respectively (Table S1), following the trend reported for films produced from starch recovered from the potato chip industry [6]. The L* values significantly decreased from 93.1 (neat films) to a minimum of 89.7 for films containing 15% BrE (Figure 3, Table S1), indicating that the incorporation of the extract decreased the films’ lightness. In addition, the b* (yellow–blue) values significantly increased from −1.20 (neat films) to 1.39 (5% BrE), 4.00 (10% BrE), and 6.31 (15% BrE), thus revealing that the incorporation of the BrE conferred a yellowish coloration to the neat films, this being even more evident for higher BrE dosages. The calculated total color difference (ΔE) values corroborated these chromatic changes, varying from 3.21, 5.94, and 8.26 in films with 5%, 10, and 15% BrE, respectively (Table S1). Thus, the high the BrE amount, the higher the color difference of the materials. These differences can be related to the presence of melanoidins in the 50–100 kDa fraction (Table 1). This trend observed for BrE was found in potato starch-based films containing coffee silverskin, a coffee roasting industry byproduct [19]. Nevertheless, as revealed in the images, the typical transparency of the neat films was preserved for all of the BrE dosages (Figure 3). Moreover, similar a* values were observed among all of the films (Table S1), meaning that the red–green coordinate was kept constant.

#### 3.2.2. Thickness and Mechanical Properties

**Figure 4:**
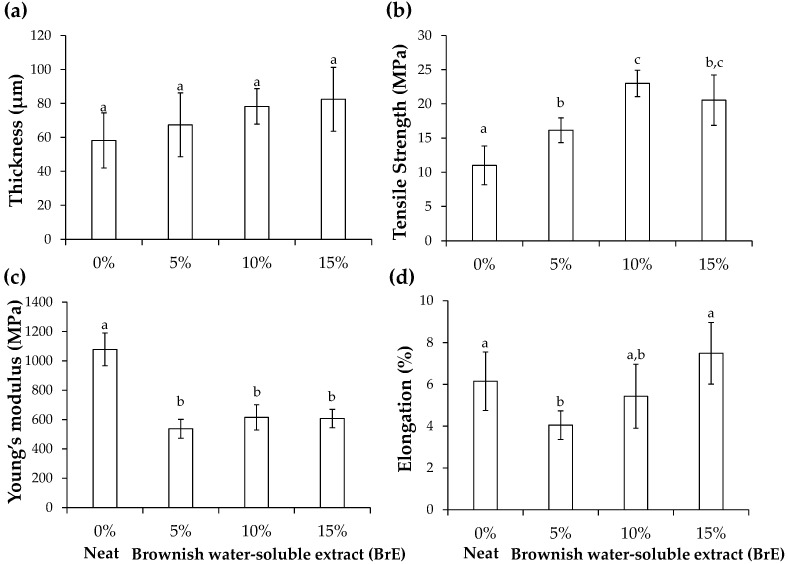
Thickness (**a**) and mechanical properties (tensile strength (**b**); Young’s modulus (**c**); elongation at break (**d**)) of potato starch-based films containing different BrE amounts (0%, 5%, 10%, and 15% *w*/*w* of dry starch weight). The values that are significantly different (*p* < 0.05) are represented by different lowercase letters.

The neat films had a thickness of ca. 58 μm and a low elongation at break (ca. 6%) and tensile strength (ca. 11 MPa), although showing a high Young’s modulus (ca. 1078 MPa) (Figure 4). These values are consistent with the brittle and rigid character of potato starch-based films [5,6,19].

Compared to the neat films, although no significant differences were observed in thickness (Figure 4a), the incorporation of BrE influenced the mechanical performance of the films. The BrE significantly increased the films’ tensile strength from ca. 11 MPa (neat films) to ca. 16 MPa, 23 MPa, and 21 MPa in films with 5%, 10%, and 15% of BrE, respectively (Figure 4b), improving the traction resistance of the materials. Additionally, BrE significantly decreased the Young’s modulus from ca. 1078 MPa (neat film) to values lower than ca. 615 MPa (Figure 4c), revealing that their elasticity increased with the incorporation of BrE, independently of its concentration. This effect can be related to the presence of BrE compounds that decrease the cohesion forces within the starch molecules, promoting a discontinuity in the polymeric matrix, similar to the trend observed with starch-based films containing spent frying oil-derived fatty acids [6]. In addition, the incorporation of 5% BrE decreased the elongation at break of the films from ca. 6% (neat film) to ca. 4%, thus decreasing their plasticity. However, for 15% BrE, the elongation at break increased to ca. 8% (Figure 4d). This increase may be justified by the presence of a higher amount of low molecular weight compounds (39.9% of <5 kDa materials, Figure S3), such as phenolic compounds and small peptides (Table 1), which may promote a plasticizing effect due to the decrease in the hydrogen bonding within the starch network. This effect follows the trend already observed for starch-based films containing phenolic-rich extracts of potato peels [7] and hibiscus [24], as well as films with coffee silverskin [19]. Hence, according to the dosage, the BrE increased starch-based films’ resistance to break and elasticity, promoting their flexibility.

#### 3.2.3. Wettability

**Figure 5:**
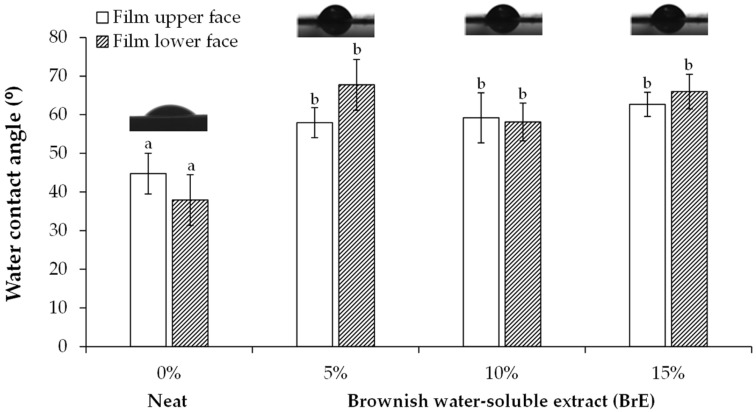
Water contact angle (WCAs) of the upper and “lower” surfaces of potato starch-based films containing different amounts of BrE (0%, 5%, 10%, and 15% *w*/*w* of dry starch weight). The values that are significantly different (*p* < 0.05) are represented by different lowercase letters.

The neat films exhibited water contact angles (WCAs) below ca. 45° at both surfaces, which highlights their hydrophilic character and homogeneous composition, following the trend that has been reported for potato starch-based materials [6,19]. When compared to the neat films, the incorporation of 5, 10, and 15% BrE increased the WCAs on both film surfaces, from ca. 45° (neat films) to ca. 58°, 59°, and 62° at the upper side and from ca. 38° (neat films) to ca. 68°, 58°, and 66° at the lower side, respectively. However, these values were neither statistically different among the three BrE dosages used nor among the two films’ surfaces (Figure 5). Therefore, BrE allowed for increasing the water tolerance of potato starch/BrE-based films. Since BrE was mainly composed of carbohydrates 66.9% *w*/*w* (Table 1), their hydroxyl groups may justify all of the WCA values below the hydrophobicity benchmark of 90°. Moreover, the presence of the 50–100 kDa brown compounds and phenolics (Table 1), may have contributed to the increase in the WCA of films containing BrE, similar to the trend observed for starch-based films containing coffee silverskin [19] and potato peel phenolic-rich extract [7]. In addition, it can be suggested that BrE can contain frying oil-derived fatty acids incorporated into the melanoidins structure, similar to what was observed for other organic acids in coffee melanoidins [10], although in insufficient amounts to promote hydrophobicity to the materials.

#### 3.2.4. Solubility and Water Vapor Permeability

**Figure 6:**
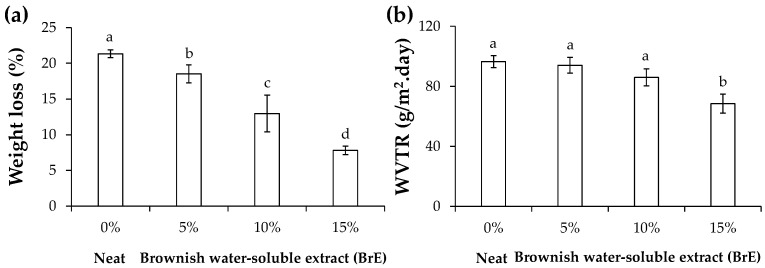
Weight loss (**a**) and water vapor transmission rate (WVTR) (**b**) of starch-based films containing different amounts of BrE (0%, 5%, 10%, and 15% *w*/*w* of dry starch weight). The values that are significantly different (*p* < 0.05) are represented by different lowercase letters.

When immersed in distilled water, the neat films (0% BrE, Figure 6a) lost their native weight in ca. 21%, which is in line with the literature on potato starch-based films [6]. This weight loss has been related to the high-water solubility of glycerol [19], the plasticizing agent. However, as a peak at 260 nm was observed in the aqueous medium of the neat films (Table S2), their weight loss can also have the contribution of leaching of the protein present in the recovered potato starch [25]. With the incorporation of BrE, the films’ weight loss significantly decreased for all BrE dosages studied (Figure 6a). These results revealed that BrE hinders the film hydration and leaching of water-soluble compounds. When compared to the aqueous medium of the neat films, a significant increase in the absorbance values at 260 nm was determined for all formulations, although not significant among the tested BrE dosages (Table S2). This indicates that the films’ weight loss can be related to the solubilization of the BrE components, possibly protein, as observed for the neat films. This suggests that the crescent incorporation of other BrE components, such as melanoidins (Table 1), avoids the glycerol bonding with water molecules and its release from the starch matrix, the efficiency of which varies linearly with the BrE concentration.

The WVTR of the potato starch-based films was ca. 97 g/m^2^·day, which is in line with the literature [19], as amylose and amylopectin from potato starch and glycerol are responsible for the passage of water vapor through the films. The incorporation of 15% BrE allowed to significantly decrease the WVTR values of the neat films to 68 g/m^2^·day. Although the other BrE dosages also tended to decrease the WVTR of the films, the values were not statistically different when compared to the neat films (Figure 6b). The highest WVTR decrease observed in films containing 15% BrE reinforces the hypothesis that BrE melanoidins (50–100 kDa, Table 1) had hydrophobic features promoted by the incorporation of frying oil-derived fatty acids, although in an insufficient amount to promote the full water vapor barrier of the materials. A similar trend was observed in starch-based films containing spent frying oil-derived fatty acids [6].

#### 3.2.5. UV-Protective and Antioxidant Properties

**Figure 7:**
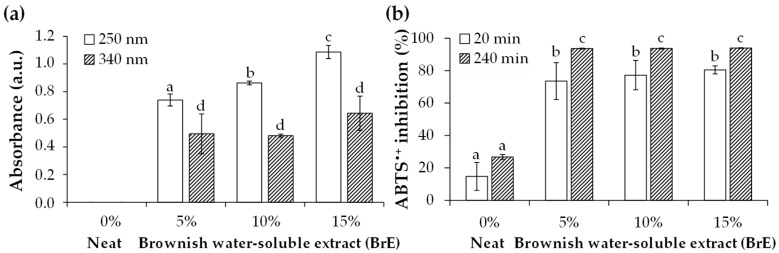
UV–visible absorption data at 250 nm and 340 nm (**a**) and antioxidant activity (by ABTS^•+^ inhibition) (**b**) of potato starch-based films containing different amounts of BrE (0%, 5%, 10%, and 15% *w*/*w* of dry starch weight). The values that are significantly different (*p* < 0.05) are represented by different lowercase letters.

From the UV–visible absorption range of 200 nm to 500 nm, two major bands at 250 nm and 340 nm were determined for films containing BrE, while no absorption was observed for the neat films (Figure 7a). This allowed for the conclusion that the BrE brown-colored components conferred a UV-protective ability, especially to the films containing 15% BrE, which had a higher melanoidins proportion (K_mix, 405 nm_ = 0.69 L/g/cm and MBI = 1.48, Table 1). In addition, the major band was registered in the UV-C region at 250 nm, the intensity of which significantly increased with the BrE dosage. A less intense band in the UV-A region, at 340 nm, was also determined, being similar for all tested BrE dosages. The protection against UV radiation may be explained by the presence of melanoidins and phenolic compounds in the 50–100 kDa BrE fraction (Table 1). Phenolic compounds from coffee silverskin have been related to the UV absorption properties of potato starch-based films [19]. This property is relevant for the application of starch/BrE-based films in food packaging for preventing food oxidation promoted by UV radiation [26].

The neat films exhibited a percentage of ABTS^•+^ inhibition of ca. 15% and 26% after 20 min and 240 min (Figure 7b). This minimal ABTS^•+^ inhibition can be explained by the presence of the phenolic compounds in the starch used to produce the films [7,27]. However, this inhibition greatly increased for films containing BrE, being similar among all tested concentrations: after 20 min and 240 min of incubation, ca. 77% and 93% of ABTS^•+^ inhibition, respectively, was achieved (Figure 7b), which may be related to the melanoidins and phenolic compounds present in the BrE (Table 1).

### 3.3. Effect of Potato Starch/BrE-Based Films on Packed Sliced Cheese Quality

The films containing 15% BrE were chosen to pack sliced cheese due to the fact of their higher water tolerance, gas barrier, UV-protective, and antioxidant properties (Figure 6 and Figure 7), while the thickness, mechanical properties, and wettability were similar to the other BrE dosages studied (Figure 4 and Figure 5).

After packaging the sliced cheese with the starch/15% BrE-based films (day 0), the appearance of wrinkles in the films promoted by direct contact with the cheese was observed. This was not observed when commercial PA/PE plastic was used (Table 2). The absorption of water molecules by the starch/15% BrE-based films was corroborated by the significant cheese weight loss: cheese packed in PA/PE material resulted in only 0.12% weight loss, while a 6.52% weight loss was observed for the cheese packed in the material containing BrE after 7 days of storage (Table 2). Accordingly, an increase in the weight of 3% was observed in the starch/15% BrE-based films. No significant differences were observed from day 7 until the end of storage, suggesting that an equilibrium in the weight lost and gain was achieved. During the storage period (14 days), the starch/15% BrE-based films wrinkles were always present. In addition, the films did not lose their adhesiveness to the cheese surface. Similar behavior was observed in starch-based films with potato peel phenolic extracts when in contact with smoked fish fillets [7].

Regarding the chromatic parameters (Table 2), on the day of packaging (day 0), the cheese samples had L*, a*, and b* values of 81.44, −2.86, and 31.04, respectively. Over time, significant changes were observed in the cheese packed in PA/PE, mainly regarding the lightness (L*) and redness (a*) coordinates (79.68 and −3.57 (day 7) and 81.74 and −2.44 (day 14), respectively), which was related to the cheese darkening. When compared to the cheese packed in the PA/PE plastics, a significant decrease in the L* values of the cheese packed with starch/15% BrE-based films was observed over the 14 days of storage (76.10 and 77.12 at day 7 and 14, respectively), indicating that the lightness of the cheese tended to decrease. In addition, a significant increase in the a* and b* values of the cheese packed with starch/15% BrE at day 14 of storage (−1.81 and 34.17, respectively) was observed, reflecting a significant decrease in the green color and an increase in the cheese yellow color. The total color difference (ΔE) values confirm the chromatic changes observed, varying from 0.52 and 2.09 in cheese packed in PA/PE and from 5.34 and 5.44 in cheese in starch/15% BrE-based films. All these chromatic changes resulted in the yellow-colored darkening of the cheese (Figure S4). The removal of water from the cheese’s surface decreases the water activity, which is a factor reported to promote Maillard reactions between lactose and lysine residues of protein when their concentration increases [28]. Nevertheless, the migration of the brown-colored compounds from the starch/BrE packaging to the cheese surface cannot be excluded, possibly due to the fact of the hydrophobic interactions with the cheese fatty acids. However, at day 7, the a* and b* coordinates of the cheese packed with the materials with BrE did not differ from the cheese at day 0; thus, the original color of the cheese was maintained over this period, evidence not observed for the cheese packed in PA/PE (Table 2).

**Figure 8:**
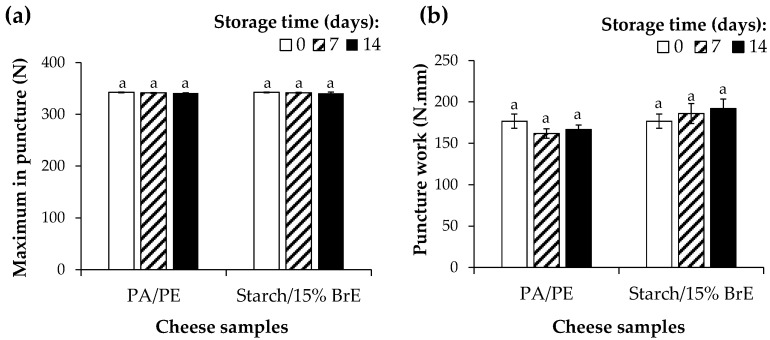
Maximum in puncture (**a**) and puncture work (**b**) of the top slices of cheese packed with starch/15% BrE-based films over 14 days of storage (4 °C, 80% relative humidity). The PA/PE-based materials are used as a reference. The values that are not significantly different (*p* > 0.05) are represented by the same lowercase letter.

The cheese texture properties are expressed in terms of the maximum in puncture (N) (i.e., the maximum force required to produce deformation) and puncture work (N.mm) (i.e., the mechanical work needed to reach the rupture point) (Figure 8). These parameters allowed to evaluate the surface hardness (cheese surface in direct contact with the packaging) and the inner hardness (cheese internal layers/paste not in contact with the packaging material) of the cheese samples, respectively [20]. Compared to the cheese before packaging (day 0) or even to cheese packed in the PA/PE materials, no significant changes were observed in terms of the maximum puncture and puncture work values for the cheese packed with starch/15% BrE-based films (Figure 8). These results revealed that both the surface and inner hardness of the cheese slices were preserved over the 14 days of storage.

Considering the volatile compounds present in the cheese samples packed with PA/PE and starch/15% BrE materials, a total of 31 compounds were determined during the 14 days of storage, belonging to the chemical families of aldehydes (7), ketones (10), alcohols (12), and acids (2) (Table S3). These chemical families were already referred in semi-hard cheese packed in poly(lactic acid) material (PLA) [29].

**Figure 9:**
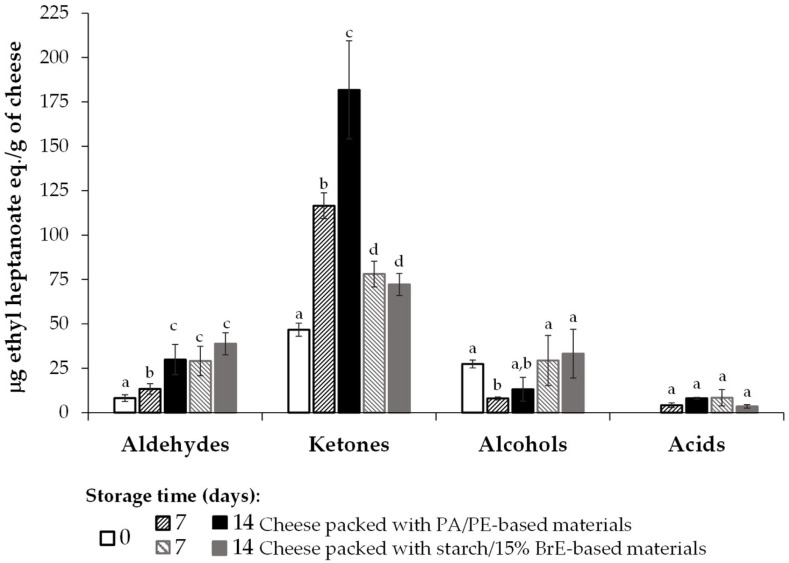
Volatile profile of the top slices of semi-hard cheese packed with starch/15% BrE-based films, stored for 14 days (4 °C, 80% relative humidity). The PA/PE-based materials are used as a reference. In each chemical family, the values that are significantly different (*p* < 0.05) are represented by different lowercase letters.

At day 0, a total of 46.72, 27.26, and 7.49 μg ethyl heptanoate eq./g of cheese of ketones, alcohols, and aldehydes, respectively, was determined in the cheese. During storage, a significant increase in the content of aldehydes was observed in cheese packed in PA/PE, mainly due to the higher increase in 3-methyl-butanal, nonanal, benzaldehyde, and benzeneacetaldehyde. A similar trend was observed for ketones, namely, 2-butanone, 2,3-butanedione, and 3-hydroxybutanone. These compounds, if present in amounts higher than their odor threshold (i.e., a lower concentration of a compound in the vapory phase that can be detected by smell [30]), can contribute with acrid, waxy, almond-like, burnt sugars, and grassy notes in the case of aldehydes and with acetone-like, butter, and woody aromas in the case of ketones [31]. Furthermore, the presence of acetic and butanoic acids was determined, which can contribute with vinegar and rancid odors [30,31]. On the other hand, a significant decrease was observed, mainly on day 7 of storage, in the amount and number of alcohols. This can be related to their oxidation, forming aldehydes, ketones, and acids, contributing to their increase in the cheese during storage (Figure 9, Table S3).

In the cheese packed in starch/15% BrE-based films, when compared to PA/PE, no significant differences were observed in the content of aldehydes and acids. However, a significantly lower concentration of ketones and a higher concentration of alcohols in the cheese were observed when stored in starch/15% BrE-based films. When compared to day 0, an increase in the aldehydes, ketones, and acids was observed, while the content of the alcohols remained similar (Figure 9, Table S3). These results indicate that BrE films can act as antioxidants, mitigating the oxidation of cheese components. In addition, over the entire storage time, no visual growth in the molds and yeasts was observed in the cheese samples, neither when the bioplastics nor when the plastics were used (images in Table 2 and Figure S4), accordingly with the absence of volatiles emitted by microbial growth.

## 4. Conclusions

The starch/15% BrE-based films revealed to be suitable for packaging semi-hard sliced cheese. They were able to preserve both the inner and surface cheese hardness over the 14 days of storage at 4 °C and 80% of relative humidity, similar to the cheese packed in PA/PE plastic. Nevertheless, a cheese weight loss and a yellow-colored darkening were observed, related to the migration of water from the cheese to the film. A similar volatile profile was also determined in the cheese packed in both materials, although the BrE constituents allowed for decreasing the content of ketones, a chemical family of compounds responsible for oxidation aromas.

The BrE, mainly constituted by carbohydrates, protein, and small amounts of phenolics, esterified fatty acids, and melanoidins, enhanced the traction resistance and promoted a plasticizing effect on the starch-based films, allowing for the development of flexible films. Additionally, the BrE decreased the films’ surface hydrophilicity and water solubility, thus enhancing their tolerance to water conditions. Moreover, the potato starch/BrE-based films possessed UV-protective and antioxidant properties, which were even more relevant for the higher BrE amount (15%, *w*/*w* in relation to starch dry weight). Therefore, the BrE was revealed to contain molecules, including melanoidins, with interest to tune the mechanical and physicochemical performances of the potato starch-based films, allowing for the development of active bio-based materials suitable for use in active food packaging applications, including cheese coating.

## Figures and Tables

**Table 1:** Yield and chemical composition of the BrE and its UF fractions.

Sample	Yield *		Ara	Gal	Glc		Total Sugars	Total Protein	Total Phenolics	Total EFA		K_mix,405 nm_		MBI
	% (*w*/*w*)		mol%				% (*w*/*w*)					(L/g/cm)		
BrE	57.2		0.95	5.20	93.9		66.9	5.70	0.40	0.10		n.d.		n.d.
>100 kDa	49.3		1.19	6.06	92.7		89.4	7.60	0.90	0.11		0.27		n.d.
50–100 kDa	3.30		2.62	12.02	85.4		29.4	17.4	7.20	0.16		0.69		1.48
<5 kDa	39.9		4.50	12.43	83.1		37.1	16.4	1.10	0.10		0.14		0.31

Ara—arabinose; Gal—galactose; Glc—glucose; EFA—esterified fatty acid; n.d.—not determined. * The BrE yield was expressed as the % of the defatted brownish frying residue, and the yield of the different molecular weight fractions is expressed as the % of the BrE.

**Table 2:** Images of sliced cheese packed in potato starch/15% BrE-based films and mean values of cheese lightness (L*), red–green (a*), and yellow–blue (b*) and total color difference (ΔE) chromatic parameters over 14 days of storage (4 °C, 80% relative humidity). The PA/PE-based materials are used as a reference.

		Sliced Cheese Samples				
		Weight Loss	Chromatic Parameters			
Image	Time (Days)	(%)	L*	a*	b*	ΔE
**PA/PE-based materials**						
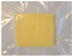	0	-	81.44 ± 0.57 ^a^	−2.86 ± 0.18 ^a^	31.04 ± 0.53 ^a^	-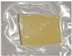	7	0.12 ± 0.04 ^a^	79.68 ± 0.50 ^b^	−3.57 ± 0.04 ^b^	30.17 ± 0.31 ^a,b^	2.09
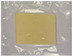	14	0.02 ± 0.03 ^a^	81.74 ± 0.91 ^a,b^	−2.44 ± 0.13 ^c^	31.12 ± 0.49 ^a^	0.52
**Starch/15% BrE-based materials**						
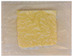	0	-	81.44 ± 0.57 ^a^	−2.86 ± 0.18 ^a^	31.04 ± 0.53 ^a^	-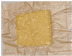	7	6.52 ± 2.16 ^b^	76.10 ± 2.01 ^c^	−2.96 ± 0.21 ^a^	30.84 ± 0.49 ^a^	5.34
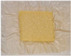	14	6.54 ± 1.58 ^b^	77.12 ± 1.99 ^b,c^	−1.81 ± 0.19 ^d^	34.17 ± 1.59 ^c^	5.44

In each column, the different lowercase letters represent values that are significantly different (*p* < 0.05).

## Data Availability

The data are contained within the article or the Supplementary Materials.

## Supplementary Materials

The following supporting information can be downloaded at: https://www.mdpi.com/article/10.3390/foods12061167/s1, Figure S1. Scheme of the experimental process to recover the brownish water-soluble extract (BrE) from potato chip industry frying residues; Figure S2. Graphic representation of the absorbance at 420, 405, 325, and 280 nm for the 5 ultrafiltration retentates (a) and for the 100 kDa (b); 50 kDa (c); 30 kDa (d); 10 kDa (e); 5 kDa (f) permeates. A total of 3 cycles were performed for each cut-off membrane (each cycle corresponding to the reduction until 50 mL of the 500 mL of sample introduced into the UF reservoir); Figure S3. Yield (in percentage) of each BrE ultrafiltration (UF) fraction. The different colors represent the chromatic tone of the fraction; Figure S4. Images of sliced cheese packed with potato starch/15% BrE-based films, highlighting the browning of the cheese surface after 14 days of storage; Table S1. Images and mean values of lightness (*L**), red–green (*a**), yellow–blue (*b**), and total color difference (ΔE) of potato starch-based films containing different amounts of BrE (0%, 5%, 10%, and 15% *w*/*w* of dry starch weight); Table S2. UV–Vis absorbance values at 260 nm of the aqueous medium of potato starch-based films containing different amounts of BrE (0%, 5%, 10%, and 15% *w*/*w* of dry starch weight), after 14 days in contact with water; Table S3. Volatile compounds, organized by chemical family, determined in the top slices of the cheese packed with potato starch/15% BrE-based materials, stored for 14 days (4 °C, 80% relative humidity). The PA/PE-based materials are used as a reference.
