# Supplementary material for: Potato Chips Byproducts as Feedstocks for Developing Active Starch-Based Films with Potential for Cheese Packaging
Source: Foods. 2023 Mar 9;12(6):1167. doi: 10.3390/foods12061167 (PMC10048258; doi:10.3390/foods12061167)
Supplement: Supplementary file 1 [file foods-12-01167-s001.zip › foods-2208949-supplementary.pdf]

Potato chips byproducts as feedstocks for developing active starch-based films with potential for cheese packaging

Ana M. Peixoto, Sílvia Petronilho*, M. Rosário Domingues, Fernando M. Nunes, Joana Lopes, Marit Kvalvåg Pettersen, Magnhild S. Grøvlen, Elin M. Wetterhus, Idalina Gonçalves and Manuel A. Coimbra

* Correspondence: silviapetronilho@ua.pt

Supplementary material

Figures captions:

Figure S1. Scheme of the experimental process to recover the brownish water-soluble extract (BrE) from potato chips industry frying residues.

Figure S2. Graphic representation of the absorbance at 420, 405, 325, and 280 nm for the 5 ultrafiltration retentates (a) and for the 100 kDa (b), 50 kDa (c), 30 kDa (d), 10 kDa (e), and 5 kDa (f) permeates. A total of 3 cycles were performed for each cut-off membrane (each cycle corresponding to the reduction till 50 mL of the 500 mL of sample introduced into the UF reservoir).

Figure S3. Yield (in percentage) of each BrE ultrafiltration (UF) fraction. Different colors represent the chromatic tone of the fraction.

Figure S4. Images of sliced cheese packed with potato starch/15% BrE-based films, highlighting the browning of cheese surface after 14 days of storage.

Tables captions:

Table S1. Images and mean values of lightness (*L**), red-green (*a**), yellow-blue (*b**), and total color difference (ΔE) of potato starch-based films containing different amounts of BrE (0%, 5%, 10%, and 15% w/w of dry starch weight).

Table S2. UV-Vis absorbance values, at 260 nm, of the aqueous medium of potato starch-based films containing different amounts of BrE (0%, 5%, 10%, and 15% w/w of dry starch weight), after 14 days in contact with water.

Table S3. Volatile compounds, organized by chemical family, determined in top slices of cheese packed with potato starch/15% BrE-based materials, stored for 14 days (4 °C, 80% relative humidity). PA/PE-based materials were used as reference.

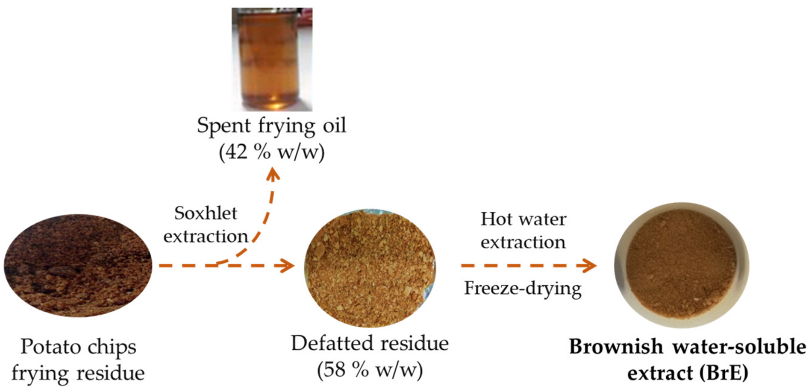

Figure S1. Scheme of the experimental process to recover the brownish water-soluble extract (BrE) from potato chips industry frying residues.

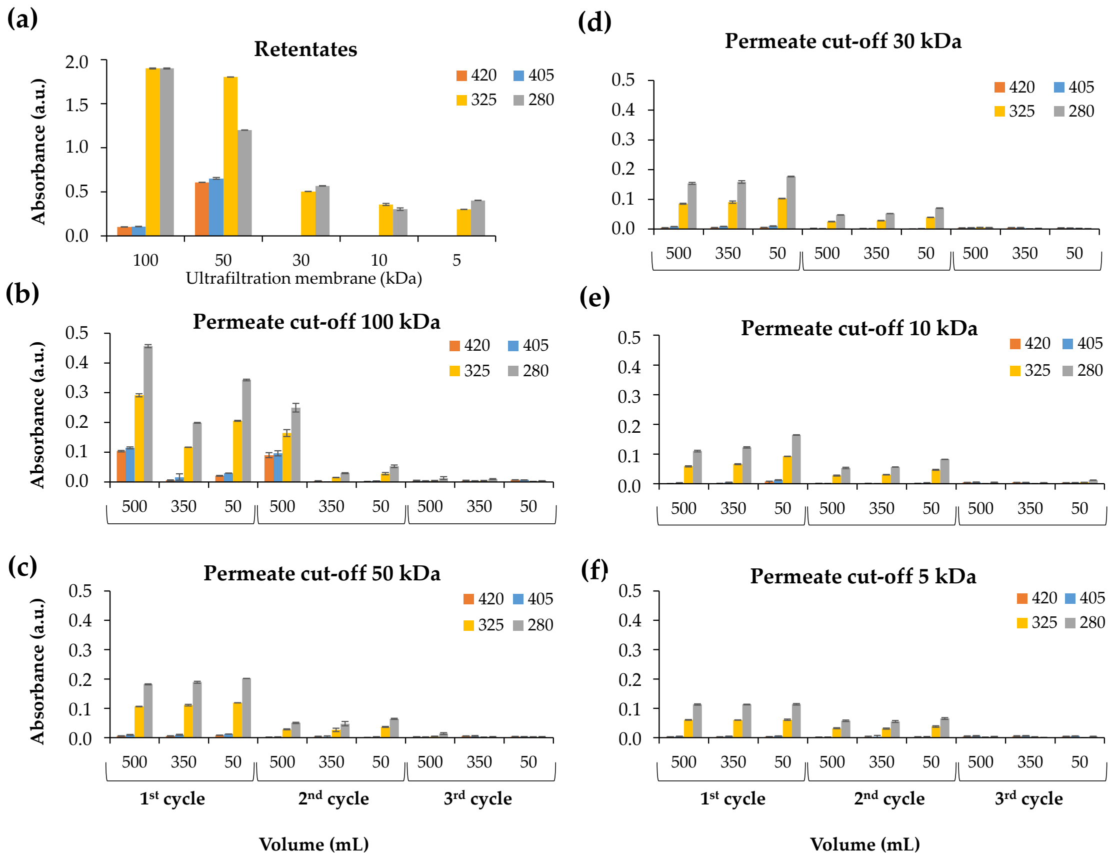

Figure S2. Graphic representation of the absorbance at 420, 405, 325, and 280 nm for the 5 ultrafiltration retentates (a) and for the 100 kDa (b), 50 kDa (c), 30 kDa (d), 10 kDa (e), and 5 kDa (f) permeates. A total of 3 cycles were performed for each cut-off membrane (each cycle corresponding to the reduction till 50 mL of the 500 mL of sample introduced into the UF reservoir).

For the 5 cut-off membranes, from the 1st cycle till the 3rd one, the UV-Vis absorbance values diminished near to zero, allowing to decide to stop the UF process. The determination of absorbances at 420 nm, 405 nm, 325 nm, and 280 nm allowed to infer about the type of compounds that composed each UF fractions. In general, it was possible to verify that all UF fractions exhibited high absorbance values at 280 nm and 325 nm, indicating that they were composed of protein and phenolics. On the other hand, mainly the 50 kDa retentate revealed higher absorbance values at 420 nm and 405 nm, suggesting the presence of high molecular weight brown compounds (melanoidins).

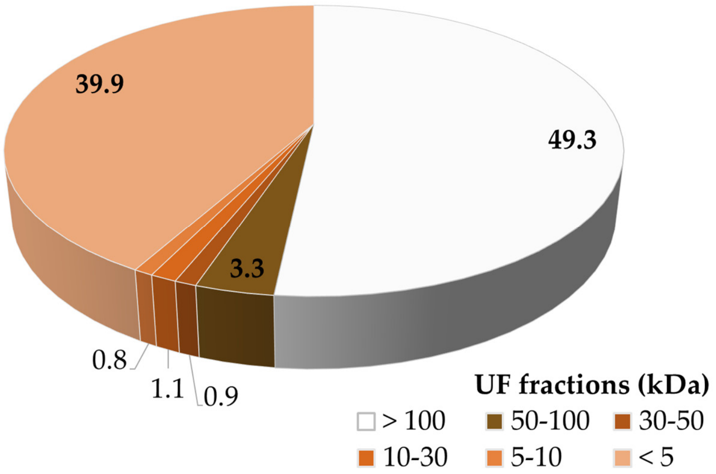

Figure S3. Yield (in percentage) of each BrE ultrafiltration (UF) fraction. Different colors represent the chromatic tone of the fraction.

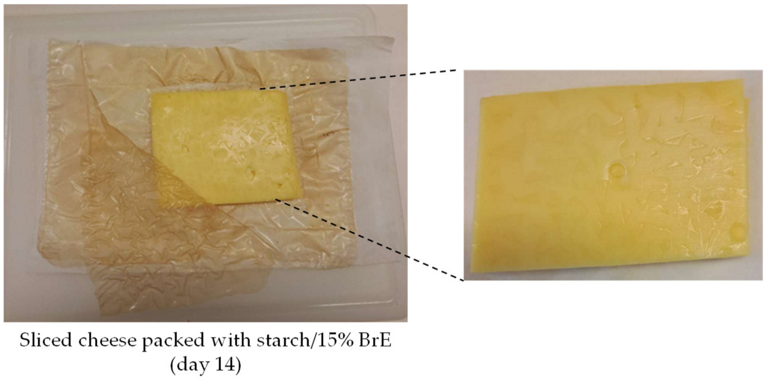

Figure S4. Images of sliced cheese packed with potato starch/15% BrE-based films, highlighting the browning of cheese surface after 14 days of storage.

Table S1. Images and mean values of lightness (*L**), red-green (*a**), yellow-blue (*b**), and total color difference (ΔE) of potato starch-based films containing different amounts of BrE (0%, 5%, 10%, and 15% w/w of dry starch weight).

Starch-based films	Real image	*L**	*a**	*b**	ΔE
0% (neat film)	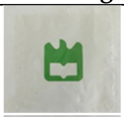	93.12 ± 0.61 ^{a}	1.73 ± 0.03 ^{a}	-1.20 ± 0.10 ^{a}	-
5% BrE	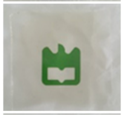	91.24 ± 0.28 ^{b}	1.53 ± 0.08 ^{a}	1.39 ± 0.57 ^{b}	3.21
10% BrE	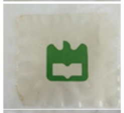	90.28 ± 0.44 ^{c}	1.31 ± 0.18 ^{a}	4.00 ± 0.93 ^{c}	5.94
15% BrE	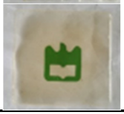	89.70 ± 0.23 ^{c}	1.44 ± 0.12 ^{a}	6.31 ± 0.69 ^{d}	8.26

In each column, different lowercase letters represent values that are significantly different ($p < 0.05$).

Table S2. UV-Vis absorbance values, at 260 nm, of the aqueous medium of potato starch-based films containing different amounts of BrE (0%, 5%, 10%, and 15% w/w of dry starch weight), after 14 days in contact with water.

Aqueous medium	Absorbance at 260 nm
0% (neat film)	0.167±0.023 ^{a}
5% BrE	0.314±0.039 ^{b}
10% BrE	0.352±0.028 ^{b}
15% BrE	0.349±0.036 ^{b}

Different lowercase letters represent values that are significantly different ($p < 0.05$).

Table S3. Volatile compounds, organized by chemical family, determined in top slices of cheese packed with potato starch/15% BrE-based materials, stored for 14 days (4 °C, 80% relative humidity). PA/PE-based materials were used as reference.

		Cheese samples (µg ethyl heptadecanoate eq./g of sample)				
		PA/PE			Starch/15% BrE	
		Storage (days)				
**Compound**	**RT (min)** [a]	**0**	**7**	**14**	**7**	**14**
Aldehydes						
2-Methyl-propanal	3.220	n.d.[b]	0.63 ± 0.03	1.64 ± 0.65	0.88 ± 0.00	1.08 ± 0.17
3-Methyl-butanal	5.954	1.30 ± 0.12	4.36 ± 0.78	10.37 ± 1.05	6.09 ± 0.37	7.36 ± 1.01
Octanal	33.318	1.70 ± 0.57	0.38 ± 0.13	0.96 ± 0.25	2.93 ± 1.17	3.55 ± 0.55
Nonanal	36.828	3.86 ± 0.85	4.44 ± 0.97	9.18 ± 1.16	12.10 ± 5.55	17.62 ± 2.85
Decanal	39.514	0.47 ± 0.16	0.46 ± 0.15	n.d.	0.78 ± 0.45	1.08 ± 0.02
Benzaldehyde	40.240	0.29 ± 0.02	0.80 ± 0.12	2.18 ± 1.39	2.33 ± 0.67	2.83 ± 0.72
Benzeneacetaldehyde	42.797	0.59 ± 0.17	2.32 ± 0.76	5.56 ± 3.99	3.98 ± 0.13	5.27 ± 0.89
Sub-total		*8.21 ± 1.89*	*13.37 ± 2.95*	*29.88 ± 8.50*	*29.09 ± 8.33*	*38.79 ± 6.21*
Ketones						
Propanone	3.289	13.89 ± 2.84	20.63 ± 3.81	31.41 ± 4.58	1.95 ± 0.42	1.33 ± 0.21
2-Butanone	5.344	17.04 ± 0.35	32.29 ± 0.14	46.27 ± 1.56	2.51 ± 0.65	1.24 ± 0.17
2-Pentanone	8.952	1.46 ± 0.07	2.67 ± 0.12	3.16 ± 1.93	n.d.	n.d.
2,3-Butanedione	9.731	5.94 ± 0.21	23.38 ± 2.35	39.18 ± 4.34	27.25 ± 1.37	31.10 ± 3.24
2-Heptanone	27.088	3.22 ± 0.11	6.35 ± 0.28	8.45 ± 5.11	4.90 ± 0.20	3.77 ± 0.08
3-Hydroxybutanone	33.117	1.24 ± 0.03	21.98 ± 0.13	38.94 ± 2.73	29.27 ± 3.43	23.50 ± 0.60
2-Nonanone	36.670	2.13 ± 0.06	5.05 ± 0.16	6.97 ± 3.83	5.15 ± 0.38	4.55 ± 0.55
2-Undecanone	41.663	1.41 ± 0.03	3.18 ± 0.14	4.64 ± 2.13	4.29 ± 0.27	4.29 ± 1.03
Acetophenone	42.926	n.d.	n.d.	1.22 ± 0.84	1.45 ± 0.28	1.42 ± 0.30
2-Tridecanone	45.640	0.39 ± 0.00	0.97 ± 0.13	1.49 ± 0.59	1.28 ± 0.21	0.98 ± 0.08
Sub-total		46.72 ± 3.70	116.51 ± 7.25	181.73 ± 27.65	78.05 ± 7.21	72.17 ± 6.25
Alcohols						
2-Butanol	12.924	21.07 ± 0.23	n.d.	n.d.	n.d.	n.d.
1-Butanol	24.522	n.d.	n.d.	n.d.	3.07 ± 0.66	3.06 ± 0.49
3-Methyl-3-buten-1-ol	31.558	0.96 ± 0.05	0.71 ± 0.24	1.32 ± 1.22	n.d.	n.d.
1-Pentanol	31.712	1.06 ± 0.00	2.27 ± 0.03	3.14 ± 1.77	2.39 ± 0.17	2.29 ± 0.34
2-Heptanol	34.524	0.83 ± 0.04	n.d.	n.d.	n.d.	n.d.
3-Methyl-2-buten-1-ol	34.620	1.57 ± 0.01	1.56 ± 0.16	2.67 ± 1.73	0.76 ± 0.76	n.d.
1-Butoxy-2-propanol	35.155	n.d.	n.d.	n.d.	2.63 ± 1.22	3.47 ± 0.16
1-Hexanol	35.615	0.50 ± 0.02	1.32 ± 0.08	2.40 ± 1.25	8.47 ± 4.08	9.75 ± 4.59
2-Butoxyethanol	37.021	n.d.	n.d.	n.d.	1.07 ± 1.07	2.66 ± 1.17
1-Heptanol	38.404	0.45 ± 0.04	1.03 ± 0.22	1.62 ± 0.64	1.75 ± 0.44	1.71 ± 0.61
2-Ethyl-1-hexanol	39.238	0.43 ± 0.14	n.d.	n.d.	6.11 ± 3.62	6.73 ± 3.45
1-Octanol	40.733	0.53 ± 0.16	1.09 ± 0.12	2.01 ± 0.07	3.13 ± 2.07	3.54 ± 1.93
Sub-Total		*27.40 ± 2.16*	*7.97 ± 0.84*	*13.16 ± 6.68*	*29.38 ± 14.09*	*33.22 ± 13.75*
Acids						
Acetic acid	38.626	n.d.	1.67 ± 0.23	2.80 ± 0.48	2.32 ± 2.32	3.51 ± 1.02
Butanoic acid	42.411	n.d.	2.52 ± 1.08	5.33 ± 0.04	6.09 ± 2.25	n.d.
Sub-Total		*0*	*4.19 ± 1.31*	*8.13 ± 0.52*	*8.41 ± 4.57*	*3.51 ± 1.02*
**Total**		**82.33 ± 7.76**	**142.04 ± 12.34**	**232.89 ± 43.35**	**144.94 ± 34.20**	**147.69 ± 27.23**

[a] RT – retention time; n.d. – not detected.
